# Supplementary material for: The implementation of a nutrition protocol in a surgical intensive care unit; a randomized controlled trial at a tertiary care hospital
Source: PLoS One. 2020 Apr 16;15(4):e0231777. doi: 10.1371/journal.pone.0231777 (PMC7162472; doi:10.1371/journal.pone.0231777)
Supplement: S1 Data — (PDF) [file pone.0231777.s005.pdf]

The implementation of Nutritional Administration Protocols in Surgical Intensive Care unit SIRIRAJ Hospital

Pornrat Chinda[1], Pulyamon Poomthong[1], Puriwat Toadithep[2], Chayanan Thanakiattiwibun[2], Onuma Chaiwat[2]*

[1]*Division of Critical Care Medicine, Department of Medicine, Faculty of Medicine, Siriraj Hospital, Mahidol University, Thailand*
[2]*Department of Anesthesiology, Faculty of Medicine, Siriraj Hospital, Mahidol University, Thailand*

Background

One of the most common problems in critically ill patients is malnutrition, which associated with an increase in morbidity and mortality. The incidence of malnutrition in hospitalization ranges between 19% and 80% worldwide. As many as 40% of adult patients are seriously malnourished at the time of their hospital admission and two third of all patients experience deterioration of their nutritional status during their hospital stay(1). A catabolic response in acute critically ill patients is much more than fasting in healthy persons; it's superimposed by an immobilization, pronounced inflammatory and endocrine stress responses. Skeletal muscle wasting and weakness are associated with a prolonged need for mechanical ventilation and rehabilitation.(2)

Nowadays in surgical intensive care unit Siriraj hospital, nutritional support is upon the judgment of attending physician teams, and some surgical patients had not reached the goal of effective nutritional supplement from various reasons, such as contraindication from surgical conditions, inadequate enteral and parenteral feeding, bowel dysfunction from stress response, etc. Critically ill surgical patients might develop poor outcomes in terms of prolonged ICU stay due to higher rates of infection, increased ventilator dependency and impaired wound healing that may be the result of malnutrition(1,3). Evidence-based guidelines help reduce evidence-practice gaps by promoting awareness of intervention of proven benefit and discouraging ineffective care(4). Barr J, et al' studied in medical-surgical ICUs of a university teaching hospital and an affiliated department of Veterans Affairs Hospital in 2004, evidence-based nutrition management protocol increased the likelihood that ICU patients would receive enteral nutrition and shortened their duration of mechanical ventilation(1). Chapman et al found that using standardized enteral nutrition orders in the ICU shortened the time to reach caloric targets in patients by 3 days(5). Spain et al showed that a tube feed infusion protocol improved the delivery of enteral tube feeds in ICU patients through improved physician ordering and more rapid advancement of tube feeds(6). However, each intensive care units are unique places, they had their policy and guidelines. Given that, Siriraj surgical-ICU researcher wants to develop our local nutritional protocol that is suitable for our surgical ICU conditions, that is the main objective to re-do the study. In addition, we would like to evaluate the efficacy of feeding and clinical outcomes of patients who are managed by a nutrition protocol.

Objectives

1. To develop a nutrition protocol in surgical Intensive Care Unit Siriraj hospital.
2. To compare the effectiveness of the nutrition therapy and clinical outcomes between the patients whose nutrition therapy are managed by the nutritional protocol group and the patients whose therapy are followed by the judgement of attending physicians (control group).

Population: Adult patients (≥18 years) admitted to surgical intensive care units (SICUs) of Siriraj Hospital.

Inclusion: Adult critically ill surgical patients who are admitted to SICUs and expected to stay in the ICUs longer than 2 days.

Exclusion:

1. Patients who are tolerating an oral diet or scheduled to return to oral intake within 24 hours.
2. Patients who are receiving palliative care, moribund and not expected to survive 6 hours.
3. Patients who are brain death or suspected brain death.
4. Patients who are admitted directly from any other ICUs.
5. Patients who are foreigners.

Study Design: Randomized Controlled trial

Methods: The study consists of 4 periods

- 2 months for protocol development
- 6months of randomized controlled trial data collection
- 2 months data analysis and interpretation

Data collection as following

1. Patient profile: age, gender, weight, height, body mass index (BMI), primary diagnosis, co-morbid disease (diabetes mellitus, hypertension, cardiovascular disease, chronic lung disease, chronic kidney disease, hematologic disease, chronic liver disease, neuropsychologic disease), smoking, alcoholic drinking, Acute Physiology and Chronic Health Evaluation II (APACHE II) score, The Sequential Organ Failure Assessment (SOFA), type and duration of surgery, presence of sepsis, history of nutritional status and weight loss.
2. Nutritional Risk Screening 2002 (NRS-2002 scores), Malnutrition Universal Screening Tool (MUST) scores and Nutrition Risk in the Critically Ill (NUTRIC) scores.

3. The time to start feeding, the percentage of patients who received EN within the first 48 hours, the total daily calories received, and the proportion of patients receiving ≥ 60% of the target calories on Day 4 of their ICU admission.
4. The percentage of patients who had hyper/hypoglycemia, vomiting, aspiration, abdominal distention, or a gastric residual volume ≥ 250 ml.
5. ICU mortality rate, the in-hospital mortality rate, the ICU/hospital LOS, the duration of mechanical ventilation, and the incidence of new infections developed in the ICU.

Surgical ICU Nutrition protocol

After enrollment, the baseline characteristics and nutritional status data were collected. The NRS-2002 and MUST scores were used to evaluate the baseline nutritional data, while the NUTRIC score was subsequently calculated to determine the need for supplemental parenteral nutrition (SPN). In the case of the protocol group, the 100%-target calories were defined as 30 kcal per kg of predicted body weight. The Siriraj SICU Nutrition Protocol (Diagram 1) was started with EN within 24 hours (with the permission of the surgeons), provided there were no contraindications (such as active shock with high-dose vasopressor; tolerating an adequate oral diet, or requiring less than 24 hours to begin an oral diet; and receiving palliative care). The acceptable surgical conditions for which EN was not allowed to commence within 24 hours are detailed in Diagram 1; those conditions were re-assessed with the surgeons every 12 hours. Although total PN might be considered for those conditions, the patients were still re-assessed for EN eligibility every 12 hours. EN was commenced with a full-strength (1:1) polymeric formula. The goal was 80% of the target calories within 72 hours. A prokinetic agent was administered if that goal was not achieved; alternatively, postpyloric feeding might be considered on a case-by-case basis. In the event that the total daily calorie intake was still < 60% of the target on Day 4 of admission, a patient’s NUTRIC score would then be redetermined and the SPN Protocol would be observed (Diagram 2). If the NUTRIC score was ≥ 5, partial PN was initiated to reach the 100% calorie-target. On the other hand, if the NUTRIC score was < 5, the EN was continued until Day 8. Partial PN was then commenced if the calories from the EN were still < 60% of the target. The management of gastrointestinal intolerance is illustrated in Diagram 3.

Diagram 1: Flow chart of Siriraj Surgical Intensive Care Unit (SICU) Nutrition Protocol.

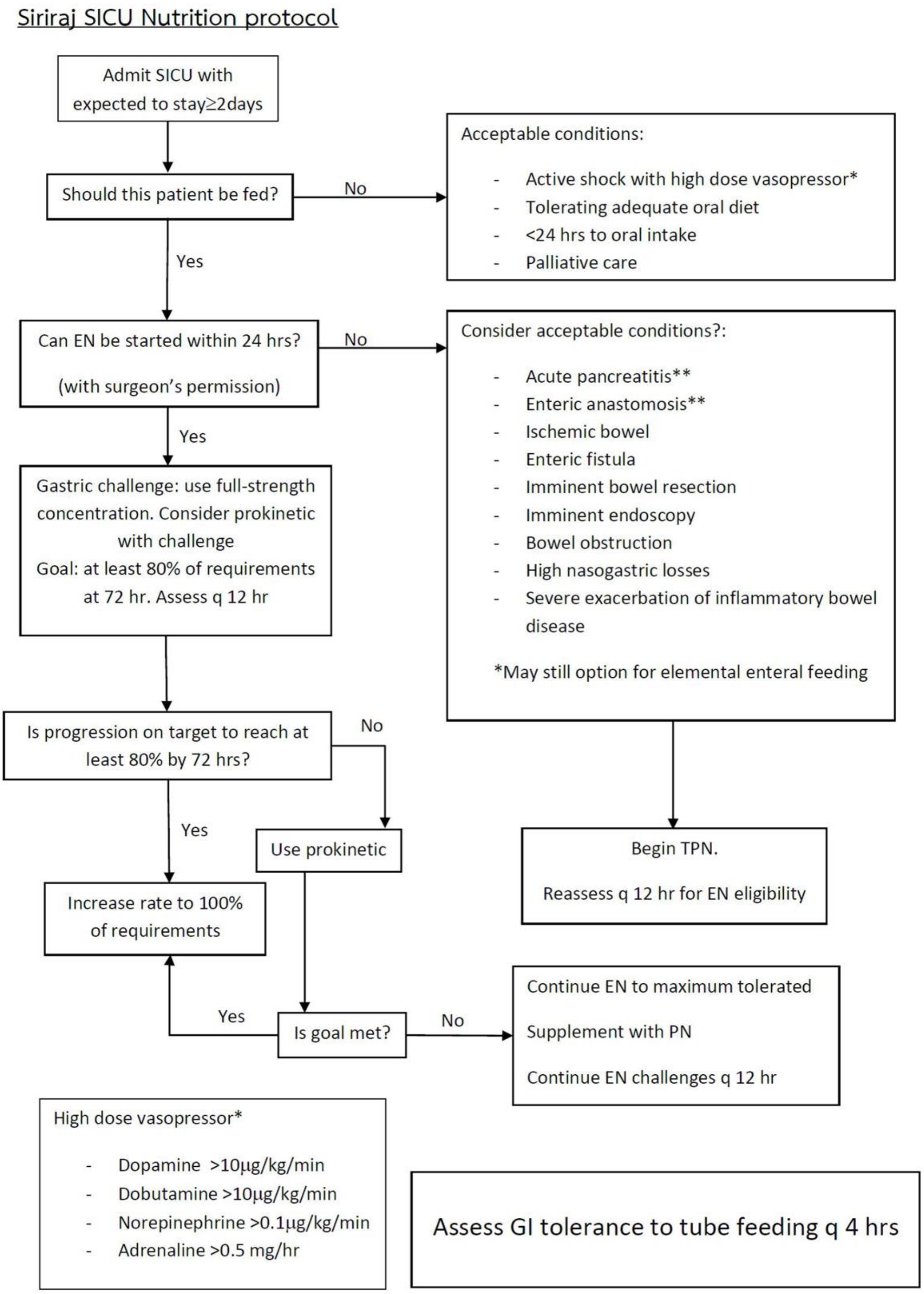

Diagram 2: Flow chart of Siriraj Supplemental Parenteral Nutrition (SPN) Protocol.

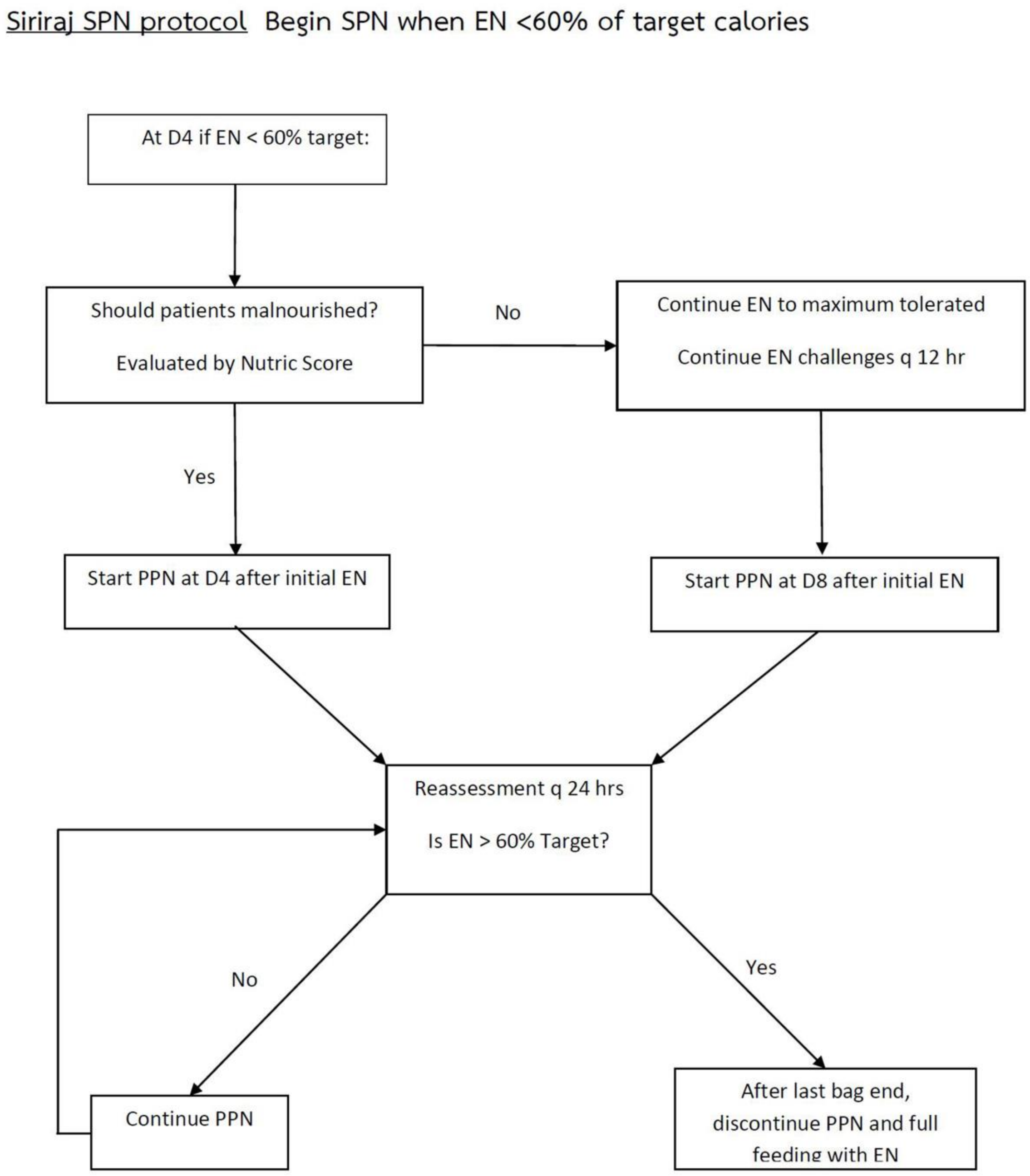

Diagram 3: Flow chart of Siriraj Gastrointestinal (GI) Intolerance Protocol.

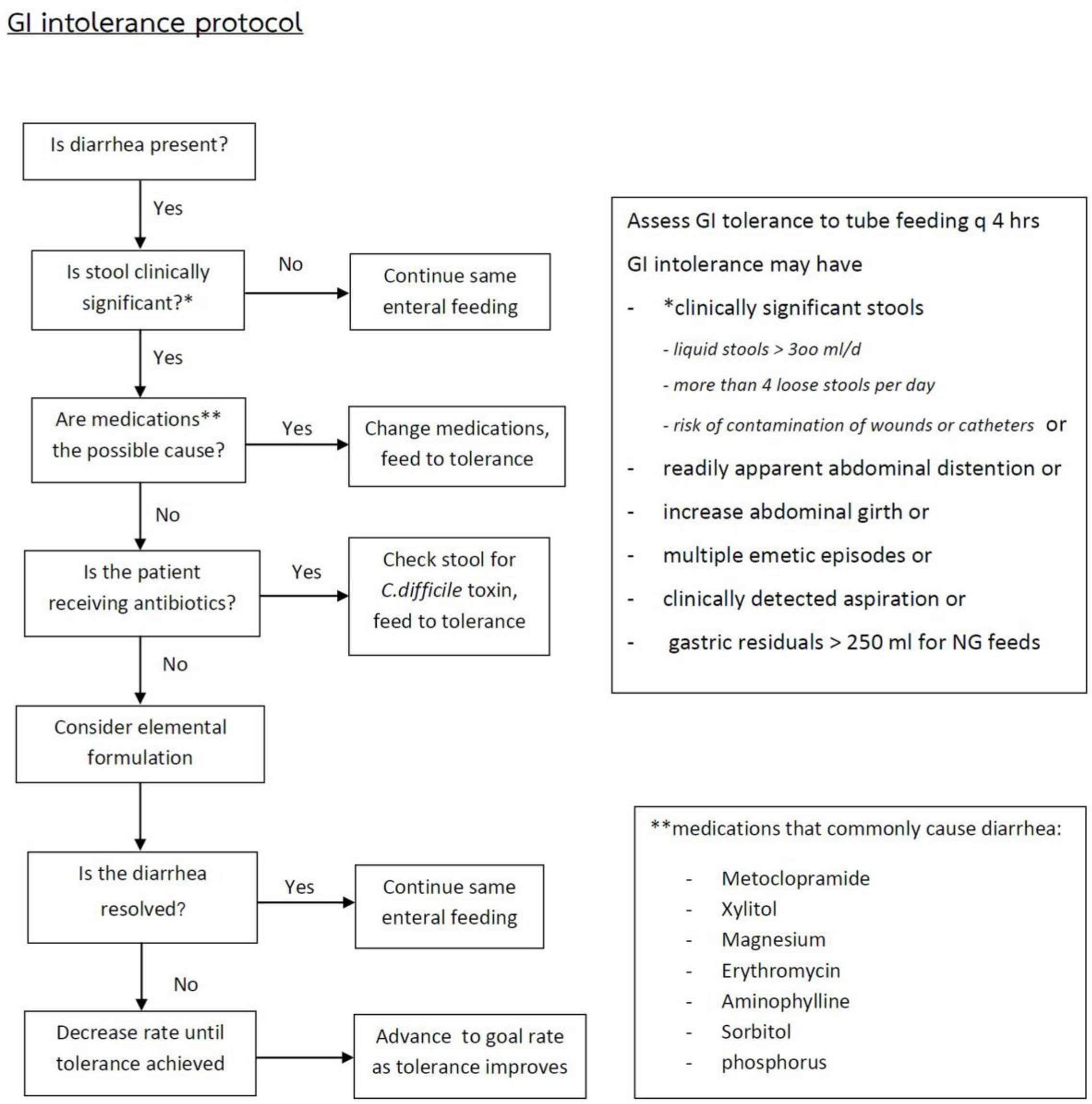

Nutritional risk screening 2002

Table 1 Initial screening		Yes	No
1.	Is BMI < 20.5?		
2.	Has the patient lost weight within the last 3 months?		
3.	Has the patient had a reduced dietary intake in the last week?		
4.	Is the patient severely ill? (e.g. in intensive therapy)		
YES: If the answer is 'Yes' to any question, the screening in table 2 is performed<br>NO: If the answer is 'No' to all questions, the patient is re-screened at weekly intervals. If the patient e.g. is scheduled for a major operation, a preventive nutritional care plan is considered to avoid the associated risk status.			

Table 2 Final screening			
**Impaired nutritional status**		**Severity of disease (≈requirement/stress metabolism)**	
**Absent<br>score 0**	Normal nutritional status	**Absent<br>score 0**	Normal nutritional requirements
**Mild<br>Score 1**	Wt loss >5% in 3 months or Food intake <50-75% of normal requirement in preceding week	**Mild<br>Score 1**	Hip fracture<br>Chronic patients, in particular with acute complications: cirrhosis, COPD, Chronic hemodialysis, diabetes, malignant oncology
**Impaired nutritional status**		**Severity of disease (≈requirement/stress metabolism)**	
**Moderate<br>Score 2**	Wt loss >5% in 2 months or BMI 18.5 -20.5 + impaired general condition or<br>Food intake < 25-50% of normal requirement in preceding week	**Moderate<br>Score 2**	Major abdominal surgery<br>Stroke<br>Severe Pneumonia, malignant hematology
**Severe<br>Score 3**	Wt loss >5% in 1 months (≈>15% in 3 months) or BMI <18.5 + impaired general condition or<br>Food intake < 0- 25% of normal requirement in preceding week	**Severe<br>Score 3**	Head injury<br>Bone marrow transplantation<br>Intensive care patients (APACHE>10)
**SCORE**	☐+	**SCORE**	☐
**Total SCORE ☐** (if age ≥ 70 add 1 score) **= ☐**			

Score ≥3: the patient is nutritionally at-risk and a nutritional care plan is initiated
Score <3: weekly rescreening of the patient. If the patient eg. Is scheduled for a major operation, a preventive nutritional care plan is considered to avoid the associated risk status

Malnutrition in critically ill patients

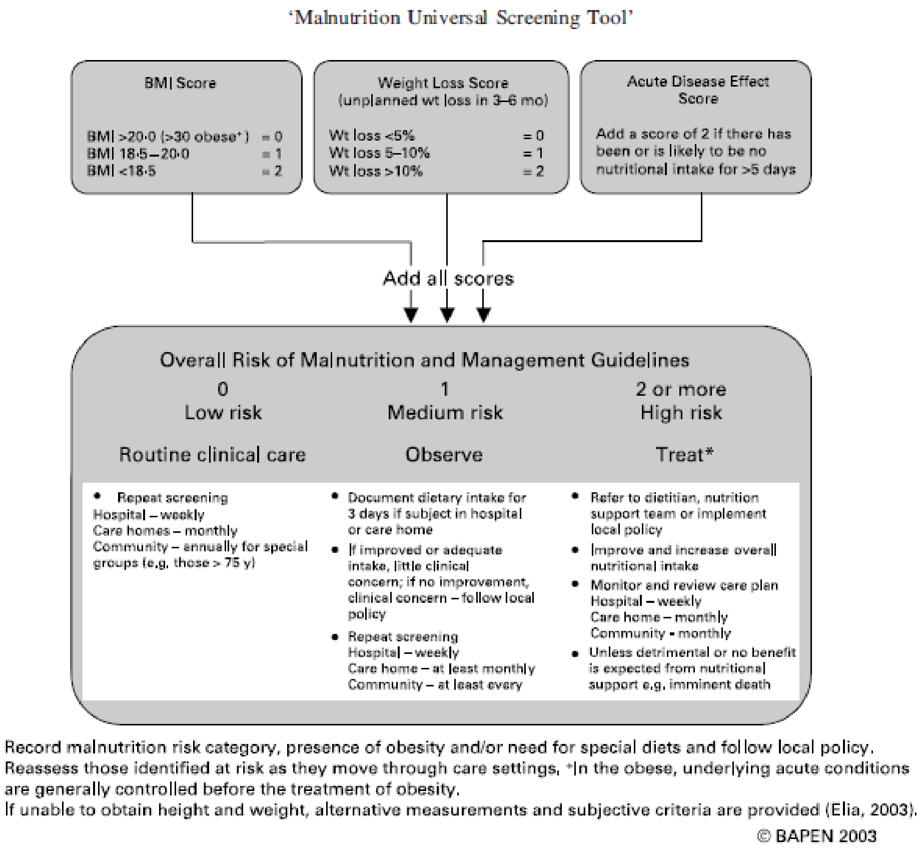

Fig. 1. 'Malnutrition Universal Screening Tool'.

Sample size calculation

To compare efficacy of nutrition administration protocols in SICU, follow to the information of old ICU Database we prognose that number of patient that met 100% target calories in control group is 50% and 75% in protocol group with statistical significant.

The output of the sample size calculation from n4Studies: for a randomized controlled trial with binary outcome.[7-9]

Formula for RCT binary data without continuity correction

$$n_{trt} = \left[\frac{z_{1-\frac{\alpha}{2}} \sqrt{\bar{p}\bar{q}\left(1+\frac{1}{r}\right)} + z_{1-\beta} \sqrt{p_1 q_1 + \frac{p_2 q_2}{r}}}{\Delta} \right]^2$$

$$p_1 = P(outcome|treatment), q_1 = 1 - p_1$$

$$p_2 = P(outcome|control), q_2 = 1 - p_2$$

$$\bar{p} = \frac{p_1 + p_2 r}{1+r}, \bar{q} = 1 - \bar{p}, r = \frac{n_{con}}{n_{trt}}$$

Formula for RCT binary data with continuity correction

$$m_{trt} = \frac{n_{trt}}{4}\left(1+\sqrt{1+\frac{2(r+1)}{n_{trt}\,r\left|p_2-p_1\right|}}\right)^2$$

$P_{control}$ = Number of surgical critically ill patient in Siriraj SICU that met 100% target calories in control group is 50%

$P_{treatment}$ = Number of surgical critically ill patient in Siriraj SICU that met 100% target calories in treatment group is 75%

P(outcome|treatment) = 0.750

P(outcome|control) = 0.500

Ratio (control/treatment) = 1.00

Alpha (α) = 0.05, Z(0.975) = 1.959964

Beta (β) = 0.10, Z(0.900) = 1.281552

So the sample size in treatments group = 77, controls group = 77 and calculated sample size by using a continuity correction treatments group = 85 and controls group= 85

Randomized population by "Block of Four" technique

Primary outcome: Efficacy of nutrition therapy provided by the SICUs compared with the non-nutrition protocol.

Secondary outcomes: ICU and hospital mortality, Hospital and ICU length of stay.

Statistical analysis of the data

- Continuous variables were expressed as means±SD or median with interquartile range (IQR).
- Categorical variables were compared using the x^2 test or fisher's exact test, as appropriate.
- Group comparisons were performed by using the independent Student's t-test, Mann–Whitney U test, chi-squared test, or Fisher's exact test, as appropriate. A two-sided alpha level of 0.05 was required for statistical significance. The data were analyzed by using the Statistical Package for Social Sciences for Windows, version 18 (SPSS Inc., Chicago, IL, USA)

Reference

1. Juliana Barr, MD, Marketa Hecht, MD, Kara E.Flavin, BA, Amparo Khorana, MSRD, CNSD, and Michael K.Gould, MD, MS, FCCP. Outcomes in critically ill patients before and after the implementation of an evidence-based nutritional management protocol. CHEST. 2004; 125(4):1446-1456.
2. De Jonghe B B-GS, Durand MC, et al. Respiratory weakness is associated with limb weakness and delayed weaning in critical illness. Crit Care Med. 2007;35:2007-15.
3. Michael P. Casaer GVdB. Nutrition in the acute phase of critical illness. N Engl J Med. 2014;370:1227-36.
4. Doig GS, Simpson F, Finfer S, et al; Nutrition Guidelines Investigators of the ANZICS Clinical Trials Group. Effect of evidence-based feeding guidelines on mortality of critically ill adults: a cluster randomized controlled trial. JAMA. 2008; 300(23):2731-2741.
5. Chapman G, Curtas S, Meguid M. Standardized enteral orders attain caloric goals sooner: a prospective study. JPEN J Parenter Enteral Nutr. 1992; 16; 149-191.
6. Spain DA, McClave SA, Sexton LK, et al. Infusion protocol improves delievery of enteral tube feeding in the critica care unit. JPEN J Parenter Enteral Nutr. 1999; 23:288-292.
7. Bernard, R. (2000). Fundamentals of biostatistics (5th ed.). Duxbery: Thomson learning, 384-385.
8. Fleiss, J. L., Levin, B., Paik, M. C. (2003). Statistical methods for rates and proportions (3rd ed.). John Wiley&Sons, 76.
9. Ngamjarus C., Chongsuvivatwong V. (2014). n4Studies: Sample size and power calculations for iOS. The Royal Golden Jubilee Ph.D. Program - The Thailand Research Fund&Prince of Songkla University.

2 WANGLANG Rd. BANGKOKNOI
BANGKOK 10700

Tel. +66 2419 2667-72
Fax. +66 2411 0162

Siriraj Institutional Review Board

Certificate of Approval

COA no. Si 618/2015

Protocol Title : The implementation of Nutritional Administration Protocols in Surgical Intensive Care Unit Siriraj Hospital

Protocol number : 332/2558(EC2)

Principal Investigator/Affiliation : Assoc. Prof. Onuma Chaiwat, M.D. / Department of Anesthesiology Faculty of Medicine Siriraj Hospital, Mahidol University

Research site : Faculty of Medicine Siriraj Hospital

Approval includes :

1. SIRB Submission form
2. Participant information sheet
3. Informed consent form
4. Nutritional risk screening 2002
5. Nutritional Protocal Case record form
6. Curriculum vitae

Approval date : November 3, 2015

Expired date : November 2, 2016

This is to certify that Siriraj Institutional Review Board is in full Compliance with international guidelines for human research protection such as the Declaration of Helsinki, the Belmont Report, CIOMS Guidelines and the International Conference on Harmonization in Good Clinical Practice (ICH-GCP).

- 9 NOV 2015

(Prof. Jarupim Soongswang, M.D.) date

Chairperson

10 NOV 2015

(Prof. Prasit Watanapa, M.D.,Ph.D.) date

Dean of Faculty of Medicine Siriraj Hospital

หน่วยพิมพ์โรงพยาบาลศิริราช 2341/3,000 แผ่น/ต.ค.57/M/2667/Mat.10023252/3060

All Siriraj Institutional Review Board Approved Investigators must comply with the Following :

1. Conduct the research as required by the Protocol ;
2. Use only the Consent Form bearing the Siriraj Institutional Review Board "APPROVED" stamp ;
3. Report to Siriraj Institutional Review Board all of serious illness of any study subject ;
4. Promptly report to Siriraj Institutional Review Board any new information that may adversely affect the safety of the subjects or the conduct of the trial ;
5. Provide reports to Siriraj Institutional Review Board concerning the progress of the research, when requested ;
6. Conduct the informed consent process without coercion or undue influence, and provide the potential subject sufficient opportunity to consider whether or not to participate.

2 WANG LANG Rd. BANGKOKNOI
BANGKOK 10700

Tel. +66 2419 2667-72
Fax. +66 2411 0162

Siriraj Institutional Review Board
Certificate of Approval (Renewal)

COA no. Si 618/2015

Protocol Title : The implementation of Nutritional Administration Protocols in Surgical Intensive Care Unit Siriraj Hospital

Protocol number : 332/2558(EC2)

Principal Investigator/Affiliation : Assoc. Prof. Onuma Chaiwat, M.D. / Department of Anesthesiology Faculty of Medicine Siriraj Hospital, Mahidol University

Research site : Faculty of Medicine Siriraj Hospital

Approval includes :

1. SIRB Submission Form Amendment 1, dated 31 Aug 2016
2. Participant Information Sheet Amendment 1, dated 31 Aug 2016
3. Informed Consent Form Amendment 1, dated 31 Aug 2016
4. Nutritional risk screening 2002
5. Nutritional Protocal Case record form
6. Curriculum vitae

Renewal date (1st) : November 3, 2016
Expired date : November 2, 2017

This is to certify that Siriraj Institutional Review Board is in full compliance with International Guidelines For Human Research Protection such as the Declaration of Helsinki, the Belmont Report, CIOMS Guidelines and the International Conference on Harmonizationin Good Clinical Practice (ICH-GCP)

(Prof. Chairat Shayakul, M.D.)
Chairperson

14 NOV 2016
date

(Prof.Dr. Prasit Watanapa, M.D., Ph.D.)
Dean of Faculty of Medicine Siriraj Hospital

14 NOV 2016
date

All Siriraj Institutional Review Board (SIRB) Approved Investigators must comply with the following:

1. Conduct the research as approved by the SIRB and will not make any changes in the research without prior SIRB review and approval, except when necessary to eliminate apparent immediate hazards to participants.
2. Use only the forms bearing the 'SIRB APPROVED STAMP' in the research.
3. Conduct the informed consent process without coercion or undue influence, and give sufficient opportunity to consider participation. One copy of the consent and/or assent form must be given to the subject after it is signed.
4. Promptly report to the SIRB of any new information that may affect the safety and well-being of the subjects.
5. Report to the SIRB all serious adverse events, unanticipated problems, protocol deviation and/or violation in accordance with the SIRB policy and operating procedures.
6. Provide the progress report to the SIRB as a Continuing Review 30 days prior to the COA expiration for at least once a year from the approval date unless otherwise indicated. The Continuing Review must be used to renew approval prior to the expired date.
7. Provide the Final Report as a close-out within 30 days after the research is complete.

Non-compliance may result in the suspension or termination of the study.
